# α-Synuclein mutations cluster around a putative protein loop^☆^

**DOI:** 10.1016/j.neulet.2013.04.058

**Published:** 2013-06-24

**Authors:** Eleanna Kara, Patrick A. Lewis, Helen Ling, Christos Proukakis, Henry Houlden, John Hardy

**Affiliations:** a Reta Lila Weston Research Laboratories, Department of Molecular Neuroscience, UCL Institute of Neurology, London WC1N 3BG, UK; b School of Pharmacy, University of Reading, Whiteknights, Reading RG6 6AP, UK; c Department of Clinical Neuroscience, Institute of Neurology, London NW3 2PF, UK

**Keywords:** α-Synuclein, SNCA, Genetics, Parkinson's disease

## Abstract

With the recent identification of two new pathogenic mutations in α-synuclein, we map the five known pathogenic mutations onto the best available models of the protein structure. We show that four of the five mutations map to a potential fold in the protein with the exception being the A30P mutation in which the substitution would be expected to have a profound effect on protein structure. We discuss this localisation in terms of the proposed mechanisms for mutation pathogenicity.

## Highlights

• We map all five missense *SNCA* mutations on the proposed α-synuclein protein models.
• 4 mutations cluster around the protein loop linking the two legs of the hairpin.
• 4 mutations cluster around the point of hairpin convergence for tetramer formation.

Mutations in α-synuclein (*SNCA*) are a rare cause of autosomal dominant Parkinson's disease (PD) accounting for a small proportion of familial cases [20]. To date, whole gene multiplications have been discovered to cause the disease [6,19,37], together with a few missense mutations. These missense mutations include A53T [34], A30P [29] and E46K [43]. Additionally, we and others have recently described two new mutations in PD cases: H50Q [1,35] and G51D [27,30]. The mechanism with which these *SNCA* point mutations initiate the disease cascade remains unknown, in contrast to whole gene multiplications which are likely to cause disease simply through increased production of α-synuclein [14]. Two possible hypotheses have been proposed to explain the pathogenicity of point mutations mainly drawing from evidence provided from pathological studies [5,28,31], studies on cell and mouse models [9,42], and most recently from *in vitro* biophysical studies [7,18]. These are the “permissive templating” hypothesis [23,26] and the “autophagy impairment” hypothesis [32,36].

The tertiary and quaternary structures formed by α-synuclein, which could provide some insight into the pathogenic mechanism associated to point mutations, remain a subject of debate [4,10,17,21]. Some evidence suggests that α-synuclein consists of two antiparallel α-helices linked through a short protein chain [39] that naturally assembles into tetramers presumably preventing α-synuclein monomer aggregation [2,13,38,41]. Though conclusive evidence has not been found, this suggestion may provide insight into the pathogenesis of PD [2]. The recent identification of the new mutations located at residues 50 [1,35] and 51 [27,30], closely adjacent to the original Contursi kindred mutation at codon 53 [34] prompted us to map them on the putative tertiary structure of the α-synuclein molecule suggested by Ulmer et al. (http://dx.doi.org/10.2210/pdb1xq8/pdb) [39] in an attempt to gain further insight into regions that are potentially crucial for the molecule's pathogenic potential. We were stimulated to do this by the clear evidence that the distribution of pathogenic mutations in amyloid precursor protein (APP) and presenilin in Alzheimer's disease gives insight into their mode of pathogenicity [24,25].

**Fig. 1:**
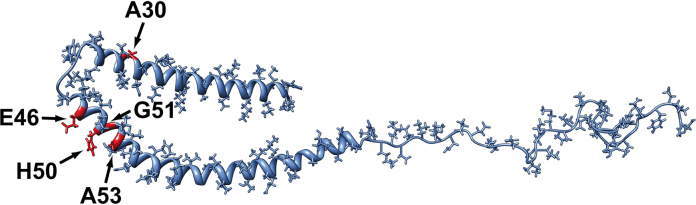
Tertiary structure for α-synuclein based on the mode proposed by Ulmer et al. [39]. The location of all missense mutations is depicted in red. Image generated using the chimera modelling program (http://www.cgl.ucsf.edu/chimera/) using PDB data for α-synuclein reference 1XQB. (For interpretation of the references to colour in figure legend, the reader is referred to the web version of the article.)

Clearly, all 5 *SNCA* point mutations cluster in proximity to the short protein loop connecting the two α-helices, with 3 mutations located on the longer arm of the α-synuclein molecule and 1 on the shorter in roughly mirroring positions, and with the E46K, H50Q, and A53T mutations aligning across the exterior of the second a-helix [8] (Fig. 1). Although the A30P mutation is further away from this loop, the effect of the proline amino acid substitution has the potential to cause greater disruption to the folding of α-synuclein than the other amino acid substitutions.

**Fig. 2:**
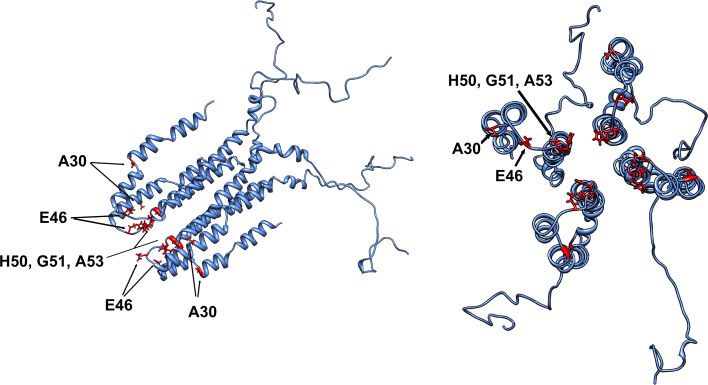
Model structure for α-synuclein modified from published solution NMR data [41] in two different projections. Ribbon presentation of an α-synuclein tetramer with disease-associated amino acid locations in red, presented as stick models. (For interpretation of the references to colour in figure legend, the reader is referred to the web version of the article.)

*Source*: Courtesy of T. Pochapsky (Brandeis), T. Bartels and D. Selkoe (Harvard Medical School).

This clustering of mutations close to the protein loop in combination with the very limited benign sequence variability (Exome Variant Server, NHLBI ESP, Seattle, WA [URL: evs.gs.washington.edu/EVS/] [accessed on 10/2012]) and associated tolerance to conformational variations of α-synuclein indicate the importance of this region and consecutively of the hairpin formation [39] for the molecule's function. Our observation would also be consistent with the suggested tetramer model for α-synuclein (Fig. 2). The hairpin structure appears to be critical for the establishment of appropriate intermolecular interactions for the tetramer formation [41]; we speculate that point mutations disrupting this hairpin conformation directly disrupt the inherently fragile tetramer and make the freely floating α-synuclein monomers susceptible to oligomerisation and aggregation, with a concomitant involvement of the proteasomal degradation system [9,42]. Thus, point mutations and whole gene multiplications could cause PD through 2 distinct mechanisms (impaired oligomer sequestration into protein tetramers in the former and increased template production in the latter case) with the same endpoint: the formation of α-synuclein aggregates in the form of Lewy bodies and Lewy neurites.

The clustering of three mutation sites in the region of four histidines with metal binding properties [11] within the core of the putative tetramer (Fig. 2) could indicate the presence of a binding pocket. Studies of soluble α-synuclein monomers have demonstrated that these associate to Cu^2+^ through their N-terminal two residues [12] and the codon 50 harbouring the sole histidine residue in the open reading frame of α-synuclein [3,15,16,35] with folding of the protein around Cu^2+^ seemingly disrupted by the H50Q mutation [35]. As no studies have been conducted on tetramer binding of small molecules/protein interactors to date, it might be the case that the nature of these interactions is altered in the context of the tetramer. Thus, further adding to our suggested model, it is possible that disruption of such small molecule binding by familial PD *SNCA* mutations could contribute to the instability of the tetramer.

**Fig. 3:**
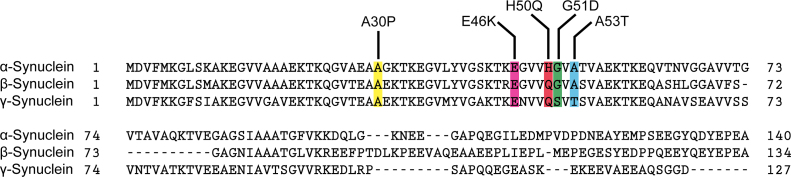
Conservation diagram of human α, β and γ synuclein molecules depicting the location of the five known missense mutations. Sequence alignment was carried out using the basic local alignment search tool (BLAST, http://blast.ncbi.nlm.nih.gov).

The variability in clinical presentation and pathology associated to each specific mutation could correspond to the degree of the hairpin and tetramer disruption and thus quantity of α-synuclein monomers available for aggregation; if this is indeed the case, the G51D mutation represents the most deleterious of all 5 mutations resulting in a disease reminiscent of the one caused by the whole gene triplication [22,27,33], whereas the A30P mutation (interestingly the only one located on the short helix) is the most “benign” [20,29]. Even though the H50Q mutation is adjacent to the severe G51D and A53T mutations, the associated clinical presentation is equivalent to the more mild phenotype associated with the A30P mutation [35]; although our proposed model does not offer a satisfactory explanation for this discrepancy, the location of residue H50 at the start of the second a-helix [41] could be relevant. It is also possible that employment of alternative metals such as zinc (which can weakly bind to H50) [40] to compensate for this codon substitution could attenuate the clinical features associated to this mutation. This hypothesis could also explain the apparent paradox presented by the fact that some of these critical residues, including the A53 residue, are not well conserved, as a substantial proportion of higher order mammals carry the human disease causing residue T in codon 53 (though the lack of conservation could also be attributed to lifespan differences between species). Similarly, codons 50, 51 and 53 are not well conserved between α, β and γ synuclein which are otherwise highly homologous molecules (Fig. 3) thus again raising the possibility of metal binding differences. Alternatively, conformational differences or the absence of such elegant tertiary and/or quaternary SNCA structures in other species and/or homologue molecules could explain these discrepancies. Clearly, the key to the understanding of the pathogenetic mechanism and significance of these point mutations would be the elucidation of the tertiary and quaternary structure of α-synuclein both in humans and other species.
